# Perforant Path Fiber Loss Results in Mnemonic Discrimination Task Deficits in Young Rats

**DOI:** 10.3389/fnsys.2018.00061

**Published:** 2018-12-11

**Authors:** Sara N. Burke, Sean M. Turner, Courtney L. Desrosiers, Sarah A. Johnson, Andrew P. Maurer

**Affiliations:** ^1^Department of Neuroscience, McKnight Brain Institute, University of Florida, Gainesville, FL, United States; ^2^Institute on Aging, University of Florida, Gainesville, FL, United States; ^3^Engineering School of Sustainable Infrastructure and Environment, University of Florida, Gainesville, FL, United States; ^4^Department of Biomedical Engineering, University of Florida, Gainesville, FL, United States

**Keywords:** cognitive aging, entorhinal cortex, hippocampus, parahippocampal region, pattern separation

## Abstract

The observation that entorhinal input to the hippocampus declines in old age is well established across human studies and in animal models. This loss of perforant path fibers is exaggerated in individuals with episodic memory deficits and Mild Cognitive Impairment, suggesting that perforant path integrity is associated with progression to Alzheimer’s Disease. During normal aging, behaviors that measure the ability of a study participant to discriminate between stimuli that share features is particularly sensitive to perforant fiber loss. Evidence linking perforant path changes to cognitive decline, however, has been largely correlational. Thus, the current study tested the causative role of perforant path fiber loss in behavioral decline by performing a unilateral knife cut to disconnect the entorhinal cortex from the hippocampus in the right hemisphere in young male and female rats. This approach does not completely disconnect the hippocampus from the entorhinal cortex but rather reduces the effective connectivity between these two structures. Male and female rats were then tested on the rodent variant of the mnemonic discrimination task, which is believed to critically rely on perforant path fiber integrity. Right hemisphere perforant path transections produced a significant impairment in the abilities of lesioned animals to discriminate between objects with high levels of feature overlap. This deficit was not observed in the male and female sham groups that received a cut to cortex above the white matter. Together these data support the view that, across species, age-related perforant path fiber loss produces behavioral deficits in the ability to discriminate between stimuli with perceptual overlap.

## Introduction

Cortical input from the parahippocampal region to the hippocampus through perforant path fibers is critical for normal cognitive functioning and is particularly vulnerable in aging and the early stages of Alzheimer’s disease. In fact, electron microscopy (Geinisman et al., 1992), electrophysiological (Barnes and McNaughton, 1980), and imaging data (Stoub et al., 2005; Yassa et al., 2010b; Rogalski et al., 2012) across animal models and humans studies have unequivocally established that there is a loss of perforant path fibers in the absence of a significant decline in entorhinal, perirhinal, or postrhinal cortical cell number with age (Rapp et al., 2002). The loss of perforant path fibers is exacerbated by Alzheimer’s disease and Mild Cognitive Impairment (Gómez-Isla et al., 1996; Kordower et al., 2001; Stoub et al., 2005, 2006), suggesting that in the early stages, these are cortical disconnection disorders (Hyman et al., 1984; Stoub et al., 2006; Leal and Yassa, 2013). Moreover, recent longitudinal data have suggested that loss of parahippocampal white matter is predictive of Alzheimer’s disease risk (Stoub et al., 2014).

A number of behaviors are disrupted by disconnection of parahippocampal cortical structures and the hippocampus, including the association between objects and places (Jo and Lee, 2010; Barker and Warburton, 2015), spatial memory (Parron et al., 2006), and instrumental contingency degradation (Corbit et al., 2002). The fiber loss during aging, however, is not a complete ablation of cortical-hippocampal functional connectivity as with lesions or pharmacological disconnections used in previous studies. Detecting the cognitive impact of age-associated declines in parahippocampal white matter integrity therefore requires sensitive behavioral probes that can be validated across human studies and animal models.

The discrimination of a target object from a similar lure is a cognitive ability that is compromised in aged rats (Burke et al., 2011; Johnson et al., 2017), and older adults (Toner et al., 2009; Yassa et al., 2010a; Stark et al., 2013; Stark et al., 2015; Stark and Stark, 2017). Specifically, older animals are more likely than young to incorrectly identify a novel lure as familiar when the lure shares features with a familiar target (Burke et al., 2011). Importantly, age-related declines in the ability to discriminate between similar objects precedes deficits on the hippocampal-dependent Morris watermaze test of spatial memory (Johnson et al., 2017), suggesting that this behavioral paradigm may be more sensitive to detecting early cognitive change. Importantly, performance on tasks that test discrimination ability is associated with perforant path fiber integrity. Imaging data from humans have shown that the perforant path diffusion signal, which is a measure of fiber integrity, positively correlates with the study participants abilities to discriminate between stimuli with overlapping features (Yassa et al., 2011; Bennett and Stark, 2016). The integrity of the fornix, which connects the hippocampus to subcortical structures (Amaral and Witter, 1995), however, has also been shown to correlate with discrimination abilities (Bennett et al., 2015). Moreover, reductions in the mean diffusion signal of the cingulum bundle, which connects the neocortex to the parahippocampal cortex (Jones and Witter, 2007), is related to discrimination abilities (Bennett and Stark, 2016). Thus, it is unknown the extent to which global declines in overall limbic white matter integrity can account for behavioral impairment in older animals compared to a direct causal relationship between perforant path fiber loss and deficits in discrimination.

The current study tested whether experimentally induced reductions in perforant path fibers in young rats with a unilateral knife cut (Skelton, 1998), that does not result in a complete ablation of entorhinal cortical neurons, could produce impairments on the rodent version of the mnemonic discrimination task, which test rats’ abilities to select a target object over a perceptually similar lure (Johnson et al., 2017). While is has been suggested the mnemonic similarity task is a behavioral test of pattern separation, that is, a hypothesized computational process in which overlapping inputs are transformed into more dissimilar outputs (e.g., Marr, 1971; McNaughton and Morris, 1987; Rolls, 2013), we have elected to not use this framework in the current paper. It has been argued that the terminology used to describe behavioral measures of stimulus discrimination should be parsed from the computational processes that may orthogonalize similar input (Santoro, 2013). Thus, the focus of the current paper is on the relationship between perforant path fiber integrity and the ability to discriminate between two stimuli with varying degrees of perceptual overlap. As the projections from the entorhinal cortex to the hippocampus are bilateral, terminating across all subfields of the left and right hippocampi (Steward and Scoville, 1976; van Groen et al., 2003), unilateral transections of the perforant path do not abolish all entorhinal input. Moreover, following entorhinal or perforant path lesions, the remaining input extends into the vacated region in the dentate gyrus, potentially mimicking age-related changes (Steward et al., 1973, 1974; Lynch et al., 1976; Steward et al., 1976; Scheff et al., 1980; Geddes et al., 1985). This unilateral transection approach enabled a relatively subtle manipulation of fiber integrity allowing us to directly test the hypothesis that perforant path fiber loss leads to impairments in discriminating between perceptually similar stimuli.

## Materials and Methods

### Subjects

A total of 30 young (4–6 months old) Fischer 344 × Brown Norway F1 hybrid rats (11 male/19 female) from the NIA colony at Taconic Farms were used in this experiment. Rats were single-housed and maintained on a reversed 12-h light/dark cycle with behavioral experiments conducted exclusively during the dark phase. Upon arrival, animals were given 7 days to acclimate to the facility and were handled a minimum of 7 days prior to beginning behavioral experiments. The current experiments used appetitive-based reinforcements to train animals. Thus, rats were fed moist chow (that is, mash) once daily after training. Daily food allotments were titrated to keep animals at approximately 85% of their arrival weights with access to water *ad libitum*. Throughout the experiment, animal’s weights were recorded daily and their food was adjusted accordingly to maintain a consistent weight throughout the duration of the experiment. Once rats reached their 85% optimal weight, they began habituation to the testing apparatus. All procedures were in accordance with the NIH Guide for the Care and Use of Laboratory Animals and approved by the Institutional Animal Care and Use Committee at the University of Florida.

### Apparatus

**FIGURE 1:**
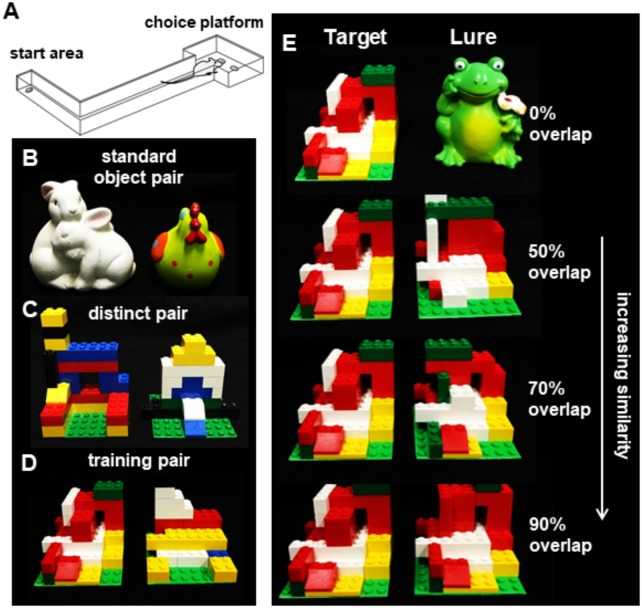
The rodent mnemonic discrimination task. **(A)** Schematic of the apparatus used for training and testing discrimination in rats. **(B)** Object pairs used for standard (no overlap) object discrimination pre-training. **(C)** Object pairs used for LEGO block object discrimination pre-training. **(D)** The LEGO block object pair used to train rats to identify the target object (left) prior to and following surgery. **(E)** The target object shown next to the (from top) 0, 50, 70, and 90% overlap lure objects.

An L-shaped track, described in detail previously (Johnson et al., 2017), was used for all object discrimination tasks. The track consisted of a start area, narrow track, and a choice platform with two food-wells (Figure 1A), was constructed out of wood and sealed with waterproof black paint. The testing room was dimly lit during experiments. The track was positioned on a table in the testing room with a desk lamp fitted with a red, 60 W light bulb placed by the starting area and an additional red light bulb illuminating the choice platform. A third lamp with a white 60 W light bulb was positioned in the corner of the room and aimed at the ceiling so that there was sufficient illumination for the rats to see objects but it was not bright as to be anxiogenic. Additionally, a webcam (Logitech; Newark, CA, United States) was positioned 50 cm above the choice platform on a boom stand to allow for video recording of each discrimination trial. Trials were recorded using a custom software interface (Collector; Burke/Maurer Laboratories, Gainesville, FL, United States). To reduce the impact of extraneous noise on behavioral performance, a white noise machine was kept on during all training and testing.

### Habituation and Shaping

Behavioral testing was carried out at least 5 days per week at approximately the same time each day. During the initial food restriction, rats were given 2–3 Froot Loops Cereal Pieces (Kellogg’s; Battle Creek, MI, United States) daily with the regular moist chow to ensure familiarity with them as a food reward. Once habituation began, a rat was placed in the start area and allowed to freely explore and retrieve Froot Loop pieces scattered throughout the track for 10 min. Depending on the animal’s propensity for exploration, habituation lasted 1–3 days and ended when the animal would explore the entire track without prompting. Next, during the shaping phase, animals were trained to alternate between the start area and choice platform (Figure 1A). Animals were positioned in the start area with a piece of Froot Loop placed pseudo-randomly in one of the two food wells of the choice platform. Animals were required to alternate between the start location and the choice platform until they could achieve 32 alternations in 20 min (Mean = 6.38 days, *SD* = 5.25).

### Standard Object Discrimination

After completing habituation and shaping, rats were trained on a distinct object discrimination problem to learn the procedural aspects of the task. Using two objects that varied in size, shape, color, and texture (Figure 1B), rats learned that when the target object was displaced, they could access a food reward in a small well under the object. The same object pair (Bunny-Rooster) was used for all rats, but the object that was rewarded was counterbalanced across rats whereby half of the rats were rewarded for Bunny and the other half rooster. To begin training, rats were placed in the start area the same as previous shaping day. The two standard objects were placed over the food wells in the choice platform with a piece of Froot Loop cereal placed under the rewarded object. The well that the rewarded object was on was alternated in a pseudo-random order over the 32 trials per session. For each trial, the rat would traverse down the arm to the choice platform containing the objects, where it would displace one of the two objects. If the correct object was chosen, the animal could access the food reward and would then traverse back to the start area where another food reward was placed. If an incorrect choice was made, the objects and Froot Loop were removed and no reward was placed at the start area. Animals completed 32 trials per day and continued training until they achieved 26 out of 32 trials (81.2%) correct. Three copies of each object were used during each test to minimize the possibility of animals using scent cues to distinguish between objects. Additionally, objects were cleaned with 70% ethanol solution between test sessions. Male rats made an average of 17 incorrect trials (±2.96 SEM) prior to reaching criterion and female rats made an average of 19 incorrect trials (±3.45 SEM), which was not statistically different [*T*_[28]_ = 0.48, *p* = 0.64].

### Distinct LEGO^®^ Block Object Discrimination and Pre-training to Target Object

After completing standard object training, rats were then tested on object discrimination using objects created out of LEGO^®^ blocks. The objects were created to be matched for overall volume and size but manipulated so that the frontal visual features differed between objects. Using a green LEGO^®^ base plate cut into 8 pips × 8 pips (6.5 cm) square, objects were constructed out of LEGO^®^ blocks on top of that base plate (Figure 1C). This object pair was distinct, sharing only 60% of pips by volume and 38% of front surface features. Following the same procedure as standard object discrimination, animals were trained that one LEGO block^®^ object was rewarded, and the rewarded object was counterbalanced across rats. As with the previous discrimination, training continued until they reached 81.2% correct (26 out of 32 trials). Female rats made an average of 48 incorrect trials (±6.53 SEM) prior to reaching criterion and male rats made an average of 57 incorrect trials (±6.06 SEM), which was not statistically different [*T*_[28]_ = 0.90, *p* = 0.38].

Once criterion performance was reached on the distinct LEGO^®^ discrimination, rats were trained following the same procedure on a novel distinct LEGO^®^ object pair. The goal of this stage is to train all animals to recognize the reward object to be presented later with novel lure objects that parametrically vary in similarity to the target. All animals were therefore trained to criterion (26 correct of 32 trials) on the same reward object (Figure 1D). Female rats made an average of 54 incorrect trials (±8.83 SEM) prior to reaching criterion, and male rats made an average of 43 incorrect trials (±9.88 SEM), which was not statistically different [*T*_[28]_ = 0.77, *p* = 0.35]. After reaching criterion, animals underwent surgical transection of the perforant pathway.

### Perforant Path Transection and Cholera Toxin Subunit B Injection

Each rat underwent stereotaxic surgery under isoflurane anesthesia (1–3%) in order to unilaterally sever the perforant path fibers of the right hemisphere and inject the retrograde tracer Cholera Toxin Subunit B in both the left (Alexa Fluor 647 Conjugate, C34778, Invitrogen, Carlsbad, CA, United States) and right (Alexa Fluor 488 Conjugate, catalog #: C22841, Invitrogen, Carlsbad, CA, United States) hemispheres. Cholera Toxin Subunit B conjugated with Alexa Fluor 488 was used to verify the knife cut, and the Alexa Fluor 647 conjugate was used as a positive control to ensure that the tracer was visible in the entorhinal cortex of the non-lesioned hemisphere. During surgery, an incision was made to expose Bregma and Lambda. A 2.5 mm-wide slot was drilled in the skull centered at 1.7 mm anterior to the intraaural line in males and extending laterally in the right hemisphere from 0.5 to 5.0 mm lateral to the midline. For females, to adjust for different in skull and brain size coordinates were adjusted based on the distance between bregma and the intraaural line (Paxinos et al., 1985). In female rats, the slot was centered at 1.9 mm anterior to the intraaural line and extending bilaterally from 0.5 to 4.75 mm lateral to the midline. Knife cuts were made using a Micro Feather Ophthalmic Scalpel No. 715 (Electron Microscopy Sciences) which had a triangular blade 7 mm long, 2 mm wide at the base and 0.2 mm thick, with an edge angled 15° to the handle. The knife was mounted on a stereotactic manipulator angled 20° from vertical in the coronal plane, so that the knife edge was angled a total of 35° from vertical. It was positioned at the correct AP coordinate (males: 4.0 mm lateral to the midline on the right side, and then inserted 5.0 mm penetrating at a 20° angle; females: 3.8 mm lateral to the midline on the right side and then inserted 4.75 mm penetrating at a 20° angle). It was then moved 2.5 mm (males) or 2.3 mm (females) medially as measured by the ‘horizontal’ scale of the manipulator, then withdrawn using the ‘vertical’ adjustment of the manipulator. The sham condition followed the same procedure as above except the blade was only inserted 1.0 mm into brain to cut only into the cortex, sparing perforant path fibers.

Immediately following the perforant path fiber transection, two additional craniotomies were drilled over the left and right hippocampi for CTB infusions. Placement of two different Alexa Fluor conjugates of this retrograde tracer (488 and 647) enabled verification of the right hemisphere perforant path fiber transection (488) with intact fiber verification in the left hemisphere (647) within the same animals. Each CTB conjugate was diluted to 1% in PBS (Conte et al., 2009a,b). All CTB infusions were made using a Nanoject II Auto-Nanoliter Injector (Drummond Scientific Company) fit with a glass pipette backfilled with the appropriate CTB conjugate. In the male rats the CTB was infused at -3.8 mm posterior to Bregma, ±2.2 mm mediolateral and between -3.4 and -2.1 ventral to the dural surface. The glass pipette was lowered to -3.4 ventral to the dural surface and 50.6 nL of CTB was infused. The pipette was left in place for 1 min and then moved up 100 μm in which another 50.6 nl infusion was placed. This process was repeated at every 100 μm until a final infusion was made at -2.1 mm ventral to dura for a total of 0.708 μL of CTB. This way the tracer infusion included the CA3 and dentate gyrus hippocampal subregions. Following the final infusion, the pipette was left in place for 150 s, and then slowly advanced up by 0.5 mm where it was left in place for another 150 s allowing for the tracer to diffuse away. After the final waiting period, the pipette was slowly removed from the brain. These infusion procedures were then repeated with other CTB conjugate in the contralateral hemisphere. The CTB infusions in the female rats were identical to those used for the males except the coordinates were adjusted to -3.6 mm posterior to bregma, ±2.1 mm mediolateral and -3.3 to -2.0 mm ventral to the dura.

### Target Retraining and Tests for Discrimination of Familiar Target From Novel Lures

Rats were allowed 1 week to recover from surgery and then placed back on food restriction and retrained to select the target object from a distinct pair of familiar LEGO objects (Figure 1D) until they returned to a criterion of 81.25% correct. After criterion performance was reached, rats were given 2 days off before their first foil test. Testing then proceeded every 3 days (day 1 test, days 2–3 off) until a total of four test sessions had been completed. This design was adopted to avoid over-familiarization with the lure objects. Each test session comprised 50 discrimination trials: 10 with a distinct control object (a frog figurine), 10 with each of 3 similar lure objects built from LEGO^®^ blocks, and 10 with an identical copy of the target object as a control. Lure objects are shown, with comparison to the target object, in Figure 1E. The most distinct Lure 1 object shared 89% volume and 50% visible features (28 of 56 bits), next Lure 2 shared 92% volume and 71% visible features (34 of 48 bits), and the most similar Lure 3 object shared 95% volume and 90% visible features (43 of 48 bits). Discrimination trials with the distinct object were included as a positive control condition, to test whether rats would continue to discriminate between pairs with no feature overlap as in initial training. Identical object discrimination trials were included to test the possibility that rats chose the target based on odor cues from the hidden food reward, or based on scent marks left on the objects on previous trials. Given that the target object was included in each of the 50 trials during each test session, 8 identical copies of this object were cycled in and out across trials. Similarly, two identical copies of each lure object were used in rotation across trials. All copies of objects were thoroughly cleaned with 70% ethanol between tests. For these tests, discrimination abilities were assessed based on percent correct responses made for each trial type. Performance was considered both for individual test sessions and collapsed as means across the four tests. Sessions were recorded, and video files were reviewed with custom software (Collector/Minion; Burke/Maurer Laboratories, Gainesville, FL, United States). Response selection behavior and reaction times were scored trial-by-trial for each test. Response selection was scored as a binary variable based on whether rats displayed ‘checking.’ Checking was defined as hesitation or pausing in front of one of the two objects prior to withdrawing attention from that object, instead switching to investigate and/or displace the alternate object. Reaction time was defined as the time elapsed between the video frame in which the tip of the rat’s nose crossed the threshold of the choice platform, and the video frame in which the rat initiated displacement of an object. During this time, the rat traversed the distance from the center point of the threshold to the center of a food well. Collector/Minion software allowed determination of reaction times for each frame of video recorded at 30 FPS, thus with precision to 33 ms.

### Verification of Perforant Path Transection

**FIGURE 2:**
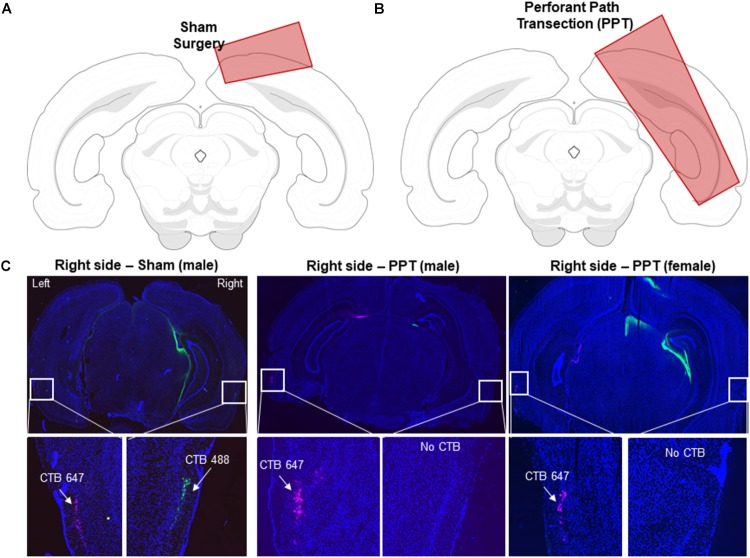
Verification of perforant path fiber transection. **(A)** Schematic of the extent of the sham knife cut through the cortex in a coronal section of the rat brain. **(B)** Schematic of the extent of the knife cut used to transect the perforant path in a coronal section of the rat brain. **(C)** Coronal sections from a control rat (left), a male PPT rat (middle) and a female PPT rat (right). Nuclei were stained with DAPI (blue). The retrograde tracer CTB was placed in the dorsal hippocampus to verify efficacy of perforant transection. CTB-647 (pink) was injected into the left hemisphere as a positive control, and CTB-488 (green) was injected into the right hemisphere. White squares indicate an area of lateral entorhinal cortex in which CTB would be present if perforant path fibers were intact. In the control Sham rats, labeling from both CTB conjugates was evident in both hemispheres. In the PPT rats, there was an absence of green labeled cell bodies of projection neurons in the right entorhinal cortex in the transected hemisphere.

Following completion of the behavioral experimental procedures, rats were placed into a bell jar containing isoflurane-saturated cotton (Abbott Laboratories, Chicago, IL, United States), separated from the animal by a wire mesh shield. Animals lost righting reflex within 30 s of being placed within the jar and immediately euthanized by rapid decapitation. Brain tissue was extracted and flash frozen in 2-methyl butane (Acros Organics, Morris Plains, NJ, United States) chilled in a bath of dry ice with 100% ethanol (-70°C). Brains were then sectioned at 20 μm, and nuclei were counterstained with DAPI (Thermo Scientific). Tissue sections were then imaged with fluorescence microscopy at 10x with a Keyence microscope (Osaka, Osaka Prefecture, Japan) to scrutinize hippocampal injection sites of CTB in both hemispheres and for CTB labeling in the entorhinal cortex. Tiled 10x images covered the full X-Y extent of tissue sections and were stitched together with automated Keyence software. All perforant path transections (PPT) were verified histologically by identifying the presence or absence of CTB488 in the right entorhinal cortex. Figure 2 schematic diagrams of the knife cut lesions used for the sham (Figure 2A) and PPT (Figure 2B) surgeries as well as representative tissue sections from a sham animal and from two rats (1 male, 1 female; Figure 2C) that were in the group that received a right side perforant pathway transection.

### Statistical Analyses

Data are presented as mean values ± 1 standard error of the mean (SEM). Analyses were performed with the Statistical Package for the Social Sciences (SPSS) v23 for Windows. Behavioral variables were compared with repeated measures ANOVAs or *t*-tests, with experimental phase or similarity of object pairs as within-subjects factors and PPT or sham surgery as a between-subjects factor. Where relevant, performance (% correct responses) was compared to chance levels (i.e., 50% correct responses) with Bonferroni-corrected one sample *t*-tests. *Post hoc* analyses were performed with simple or repeated contrasts, and a Bonferroni correction was applied when appropriate.

## Results

### Object Discrimination Testing and Post-surgical Retraining on Target Object

**FIGURE 3:**
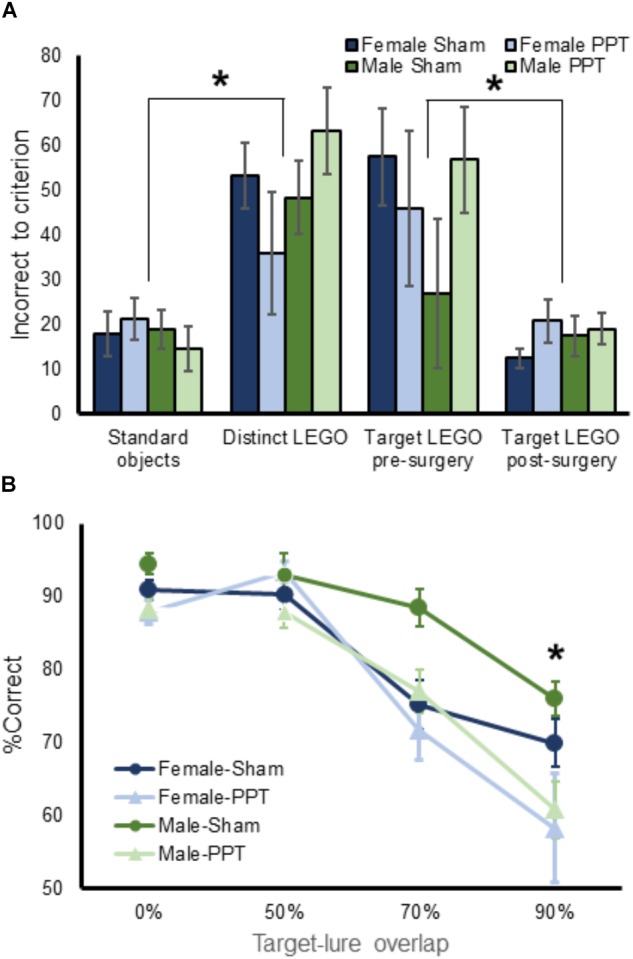
Performance on object discrimination tasks by experimental group. **(A)** Shows the mean number of incorrect trials made prior to reaching criterion performance on all pre-training object discrimination problems for female (blue) and male (green rats) in the Sham (dark) and PPT (lighter) groups. There were no significant groups differences across training problems. **(B)** Mean percent corrent on target-lure discrimination as a function of overlap. Rats performed significantly worse as target-lure similarity increased (*F*_[3,78]_ = 61.43, *p* = 0.0001). Moreover, the PPT group performed significantly worse compared to the sham group (*F*_[1,26]_ = 8.80, *p* = 0.006), and the interaction between target-lure similarity and experimental group was statistically significant (*F*_[3,78]_ = 3.02, *p* = 0.035). *Post hoc* comparison indicated that the PPT rats made significantly more errors compared to sham animals on the 90% overlap lure object discrimination (corrected α = 0.05/4 = 0.0125, *T*_[28]_ = 2.74, *p* = 0.011). There was no overall main effect of sex on performance (*F*_[1,26]_ = 2.51, *p* = 0.13). Error bars are ± 1 Standard error of the mean (SEM). ^∗^Indicates significant effect.

Figure 2 illustrates the knife cut used in the sham surgery and the PPT, as well as representative images from a sham rat and two animals that received the fiber transection. After recovery from surgery, rats were retrained to discriminate the target LEGO block object from the familiar LEGO block lure (Figure 1D). This retraining was conducted as described in the Methods. Briefly, the two LEGO block objects were placed over the food wells in the choice platform with a piece of Froot Loop cereal placed under the target object. The rat could retrieve the reward if the target was displaced. If the lure object was chosen, the animal did not get a reward and had to then traverse back to the start area to initiate another trial. Figure 3A shows the number of incorrect trials made prior to reaching criterion performance by female (blue) and male (green) rats on all object discrimination problems that were incrementally learned for the sham (darker) and PPT (lighter) groups. Repeated measures ANOVA with the within-subjects factor of object discrimination problem (standard – Figure 1B, distinct LEGO – Figure 1C, LEGO target pre-surgery and post-surgery – Figure 1D), and the between-subjects factors of sex and treatment condition detected a significant effect of object discrimination problem on the number of incorrect trials made prior to reaching criterion (*F*_[3,78]_ = 15.11, *p* = 0.0001). This main effect of object discrimination problem did not interact significantly with any of the between-subjects factors (*p* > 0.142 for all interaction terms). Difference contrasts indicated that rats made significantly more errors when learning the distinct LEGO discrimination relative to the standard object discrimination (*F*_[1,26]_ = 31.30, *p* = 0.001), which is consistent with previous data (Johnson et al., 2017). The incorrect trials made on the two LEGO object problems prior to surgery, however, did not significantly differ (*F*_[1,26]_ = 2.8, *p* = 0.11), indicating that different LEGO object discrimination problems are acquired at a similar rate, and there are no savings from one problem to the next. Interestingly, when rats were re-trained on the target LEGO object discrimination problem after surgery, they showed savings, making significantly fewer errors compared to initial training (*F*_[1,26]_ = 38.52, *p* = 0.001). This improved performance during re-training following surgery did not significantly interact with sex (*F*_[1,26]_ = 0.10, *p* = 0.76) or treatment group (*F*_[1,26]_ = 0.12, *p* = 0.73). These data show that even when the right perforant path was transected, the performances of both male and female rats benefitted from pre-surgical experience with the LEGO objects. Moreover, PPT did not produce any overt declines in the animals’ abilities to discriminate between 2 distinct LEGO block objects, suggesting that general sensorimotor function and motivation were not impacted by the fiber transection.

### Unilateral Perforant Path Transection Impairs Discrimination Performance Based on Target–Lure Similarity

Target-lure object discrimination testing (Figure 1E), required rats to select a familiar Target object from a novel LEGO object lure. Four different lure objects were used that varied in similarity to Target. An additional lure object was included that was identical to the Target, and these identical objects served as a negative control to ensure that rats were not able to smell the reward or using another latent factor to solve the problem. Performance on this problem should, therefore, be close to chance. Critically, all rats averaged 52.5% (±0.78 SEM) correct on the identical problem and performance on this problem did not significantly vary by sex (*F*_[1,26]_ = 0.23, *p* = 0.64) or condition (*F*_[1,26]_ = 0.88, *p* = 0.36), nor was the interaction term significant (*F*_[1,26]_ = 2.62, *p* = 0.12). These data indicate that both male and female rats in the sham and PPT groups were likely using the features of the objects to select the correct choice rather than an olfactory cue or another unaccounted variable.

Mean performances across the 4 days of target-lure discrimination tests were calculated for each trial type. These values are shown in Figure 3B for the different groups of male and female rats. Mean values for the 4 different trial types were entered into a repeated measures ANOVA with lure similarity (0, 50, 70, and 90%) as a within-subjects factor and sex and experimental group (sham versus PPT) as between-subjects factors. This analysis revealed a significant main effect of target-lure similarity (*F*_[3,78]_ = 61.43, *p* = 0.0001), with percentages correct decreasing as a function of increasing similarity. Difference contrasts confirmed that while performance on the unrelated lure problem (0% overlap) was not significantly different from the 50% overlap lure (*F*_[1,26]_ = 0.22, *p* = 0.64), the percentage of correct responses significantly decreased between 50 and 70% overlap (*F*_[1,26]_ = 42.65, *p* = 0.0001) and between 70 and 90% overlap (*F*_[1,26]_ = 101.48, *p* = 0.0001), confirming the relationship between declining performance with increasing target-lure similarity.

Overall, the PPT group performed significantly worse compared to the sham group (*F*_[1,26]_ = 8.80, *p* = 0.006). Moreover, the interaction between target-lure similarity and experimental group was statistically significant (*F*_[3,78]_ = 3.02, *p* = 0.035), indicating that impairments induced by the unilateral fiber transection varied across the different lure conditions. *Post hoc* comparison indicated that the PPT rats made significantly more errors compared to sham animals on the 90% overlap lure object discrimination (corrected α = 0.05/4 = 0.0125, *T*_[28]_ = 2.74, *p* = 0.011). The effect of experimental group of performance did not reach significance for the other lures (*p* < 0.025 for all other comparisons). Together these data show that the greatest impairment produced by the unilateral PPT was for the object discrimination problem that had the most similarity between the target and lure.

While there was no overall main effect of sex on performance (*F*_[1,26]_ = 2.51, *p* = 0.13), there was a trend for sex to interact with lure-target similarity (*F*_[1,26]_ = 2.26, *p* = 0.088). In fact, it is evident in Figure 3B that female sham rats had a tendency to perform worse than males on the lure 2 problem. When performances between males and females were compared across the different lure conditions with *post hoc* analyses, however, there were no statistically significant differences (corrected α = 0.05/4 = 0.0125, *p* > 0.048 for all comparisons). Moreover, the three-way interaction between target-lure similarity, sex and experimental group did not reach significance (*F*_[3,78]_ = 0.20, *p* = 0.90).

**FIGURE 4:**
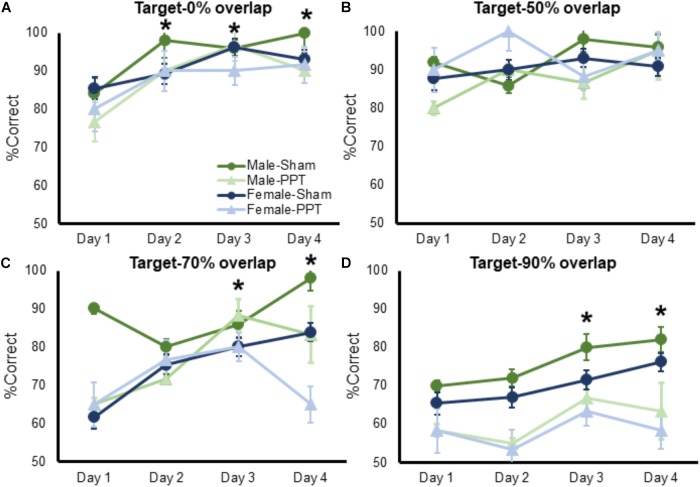
Performance on target-lure discriminations as a function of test day. In all panels mean percent corret is shown on the *Y*-axis and test day is represented on the *X*-axis. **(A)** Performance on 0% lure trials significantly improved as a function of test day (*F*_[1,26]_ = 11.73, *p* = 0.002), which did not interact with sex (*F*_[1,26]_ = 0.56, *p* = 0.65) or experimental group (*F*_[1,26]_ = 0.18, *p* = 0.91). **(B)** Performance on 50% lure trials did not significantly improve as a function of test day (Figure 4B; *F*_[1,26]_ = 2.16, *p* = 0.10), and this did not interact with sex (*F*_[1,26]_ = 1.35, *p* = 0.26). The percent of correct trials over testing days did, however, significantly interact with experimental group (*F*_[1,26]_ = 3.11, *p* = 0.031). *Post hoc* analysis showed that PPT rats improved significantly between test days 1 and 2 (corrected α = 0.05/3 = 0.017; *T*_[11]_ = 2.87, *p* = 0.015), while sham animals did not show a significant improvement across any test day (*p* > 0.14 for all comparisons), as they may have already been near ceiling performance at day 1. **(C)** Performance on 70% lure trials significantly improved as a function of test day (*F*_[1,26]_ = 5.52, *p* = 0.002), which did not interact with sex (*F*_[1,26]_ = 2.02, *p* = 0.12) or experimental group (*F*_[1,26]_ = 2.27, *p* = 0.09). **(D)** Percent of correct responses on 90% lure trials significantly improved as a function of test day (*F*_[1,26]_ = 3.89, *p* = 0.012), which did not significantly interact with sex (*F*_[1,26]_ = 0.14, *p* = 0.94) or experimental group (*F*_[1,26]_ = 0.80, *p* = 0.50). Error bars are ± 1 SEM. ^∗^Indicates significant effect.

To determine whether experience could improve an animal’s ability to discriminate between the target and a lure, percent correct responses for the 0, 50, 70, and 90% lure trials were entered into separate repeated measures ANOVAs, with day as a within-subjects factor and sex and group as the between-subjects factor (Figures 4A–D). Performance on 0% lure trials significantly improved as a function of test day (Figure 4A; *F*_[1,26]_ = 11.73, *p* = 0.002), which did not interact with sex (*F*_[1,26]_ = 0.56, *p* = 0.65) or experimental group (*F*_[1,26]_ = 0.18, *p* = 0.91). Planned comparisons showed that rats made significantly more correct responses on days 2 through 4 compared day 1 of testing (*p* < 0.002 for all comparisons). These data suggest that unilateral PPT did not impair male or female rats abilities to improve with experience at discriminating between distinct objects.

Performance on 50% lure trials did not significantly improve as a function of test day (Figure 4B; *F*_[1,26]_ = 2.16, *p* = 0.10), and this did not interact with sex (*F*_[1,26]_ = 1.35, *p* = 0.26). The percent of correct trials over testing days did, however, significantly interact with experimental group (*F*_[1,26]_ = 3.11, *p* = 0.031). *Post hoc* analysis showed that PPT rats improved significantly between test days 1 and 2 (corrected α = 0.05/3 = 0.017; *T*_[11]_ = 2.87, *p* = 0.015), while sham animals did not show a significant improvement across any test day (*p* > 0.14 for all comparisons), as they may have already been near ceiling performance at day 1. Performance on 70% lure trials significantly improved as a function of test day (Figure 4C; *F*_[1,26]_ = 5.52, *p* = 0.002), which did not interact with sex (*F*_[1,26]_ = 2.02, *p* = 0.12) or experimental group (*F*_[1,26]_ = 2.27, *p* = 0.09). Planned comparisons showed that rats made significantly more correct responses on days 3 through 4 compared day 1 of testing (*p* < 0.006 for all comparisons). Finally, the percent of correct responses on 90% lure trials significantly improved as a function of test day (Figure 4D; *F*_[1,26]_ = 3.89, *p* = 0.012), which did not significantly interact with sex (*F*_[1,26]_ = 0.14, *p* = 0.94) or experimental group (*F*_[1,26]_ = 0.80, *p* = 0.50). Planned comparisons showed that rats made significantly more correct responses on days 3 through 4 compared day 1 of testing (*p* < 0.034 for all comparisons).

### Behavioral Strategies and Reaction Times as a Function of Target-Lure Similarity

Potential sex and group differences in performance strategies used during target-lure discrimination testing were assessed by quantifying the magnitude of a response bias (that is, an animal’s tendency to select an object on a particular side, regardless of identity), reaction times, and the presence of ‘checking’ behavior (that is, pausing and sampling both objects before making a choice) (Johnson et al., 2017). The ratio of trials with checking behavior to total trials for each rat was averaged across the 4 test sessions. Within a trial, multiple checks were tallied so that it was possible for the ratio to exceed a value of 1.

**FIGURE 5:**
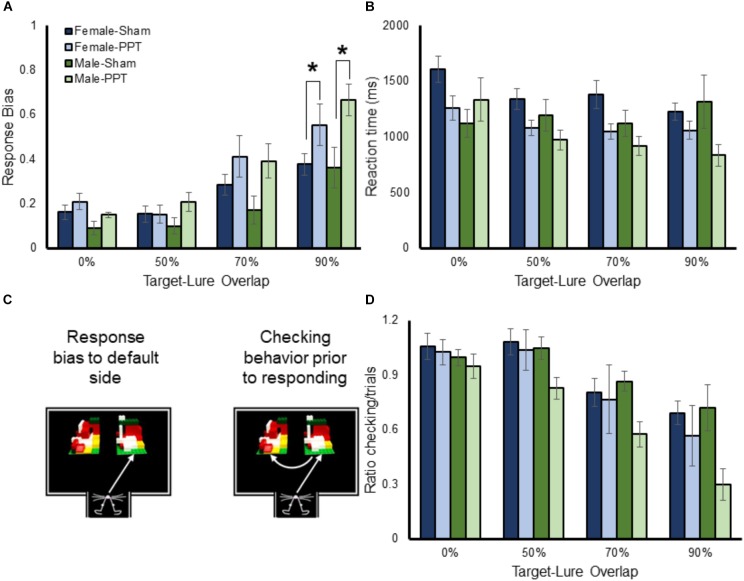
Response bias, reaction times and checking behavior as a function of target-lure similarity. **(A)** shows the mean response biases (*Y*-axis) for male and female Sham and PPT animals as a function of target-lure similarity (*X*-axis). The response bias was higher for more similar target-lure problems (*F*_[3,78]_ = 40.26, *p* = 0.0001). The main effect on target-lure similarity on response bias did not interact with sex (*F*_[3,78]_ = 1.24, *p* = 0.301). There was, however, a significant interaction with experimental group (*F*_[3,78]_ = 3.40, *p* = 0.022), due to the PPT rats having larger response biases relative to the Sham animals as the target-lure similarity increased. **(B)** Shows the reaction times (*Y*-axis) as a function of target-lure similarity (*X*-axis). Reaction times were faster for the more similar lure conditions (*F*_[3,78]_ = 6.99, *p* = 0.001). The tendency for rats to choose an object faster when the target and lure were more similar did not significantly interact with sex (*F*_[3,78]_ = 0.68, *p* = 0.57), or group (*F*_[3,78]_ = 1.92, *p* = 0.13). **(C)** Shows a schematic of checking behavior. **(D)** Shows the mean ratio of checking behavior to total trials (*Y*-axis) as a function of lure similarity (*X*-axis) for the different groups. Checking behavior significantly decreased as a function of target-lure overlap (*F*_[3,78]_ = 33.79, *p* = 0.0001). The amount of checking behavior as a function of target-lure overlap did not significantly interact with sex (*F*_[3,78]_ = 0.18, *p* = 0.91), or group (*F*_[3,78]_ = 1.78, *p* = 0.16). However, there was a trend for the PPT rats to display significantly less checking behavior compared to sham animals (*F*_[1,26]_ = 3.96, *p* = 0.057). ^∗^Indicates significant effect.

While most rats display a bias toward one side early in training, this default response must be inhibited in order for performance to improve (Lee and Byeon, 2014). In fact, previous studies have reported that a high response bias is associated with poor performance in aged rats (Hernandez et al., 2015; Johnson et al., 2017). Figure 5A shows the mean response biases for male and female Sham and PPT animals as a function of target-lure similarity. A repeated-measures ANOVA with the within-subjects factor of target-lure similarity and the between-subjects factors of sex and experimental group identified a significant main effect of similarity (*F*_[3,78]_ = 40.26, *p* = 0.0001), such that the response bias increased with the amount of overlap. Planned comparisons indicated that the response bias between the 0 and 50% overlap conditions was not significantly different (difference contrast, *p* = 0.98). However, the response bias was significantly greater for each subsequently increasing overlap between target and lure (difference contrast, *p* > 0.0001, for both comparisons). The main effect on target-lure similarity on response bias did not interact with sex (*F*_[3,78]_ = 1.24, *p* = 0.301). There was, however, a significant interaction with experimental group (*F*_[3,78]_ = 3.40, *p* = 0.022), due to the PPT rats having larger response biases relative to the Sham animals as the target-lure similarity increased. *Post hoc* comparisons detected significantly larger response biases in the PPT animals relative to Sham rats for the 90% overlap condition (*T*_[28]_ = 3.39, *p* = 0.002, corrected α = 0.05/4 = 0.0125) and a trend for the 70% overlap condition (*T*_[28]_ = 2.25, *p* = 0.033, corrected α = 0.05/4 = 0.0125), while response bias values for the 0% (*T*_[28]_ = 1.05, *p* = 0.30) and 50% (*T*_[28]_ = 0.98, *p* = 0.33) overlap problems were not significantly different between groups. Finally, the three-way interaction between target-lure similarity, sex and group did not reach statistical significance (*F*_[3,78]_ = 0.27, *p* = 0.85).

Rats that have larger response biases tend to have faster reaction times during object discrimination tasks, due to the lack of suppressing the default strategy and checking both objects (Johnson et al., 2017). Thus, the reaction times of all rats across the different target-lure discrimination problems were submitted to a repeated-measures ANOVA with the within-subjects factor of lure condition, and the between-subjects factors of sex and group. Figure 5B shows the mean reaction times for the male and female Sham and PPT rats. Consistent with previous work (Johnson et al., 2017), reaction times were faster for the more similar lure conditions that were associated with the greatest response bias (*F*_[3,78]_ = 6.99, *p* = 0.001). The tendency for rats to choose an object faster when the target and lure were more similar did not significantly interact with sex (*F*_[3,78]_ = 0.68, *p* = 0.57), or group (*F*_[3,78]_ = 1.92, *p* = 0.13). Interestingly, there was a significant three-way interaction between lure, sex and group (*F*_[3,78]_ = 5.06, *p* = 0.003). This is likely due to the relationship between decreasing reaction times and increasing similarity being evident in the female Sham and PPT rats, as well as the male PPT rats. The male sham rats, however, did not show the same pattern, and reaction times did not significantly decrease for the high overlap discrimination problems (*F*_[1,4]_ = 0.65, *p* = 0.47). These data suggest that there may be a sex difference in the evaluation of similar objects that is disrupted by the unilateral PPT.

Checking behavior (Figure 5C) is associated with slower reaction times and better performance (Johnson et al., 2017). Interestingly, it has been shown that checking behavior in rats performing an object discrimination problem is less likely to occur as the similarity between the two objects increases. This conceivably occurs because a rat will default to a response bias as the problem becomes more difficult or ambiguous. Importantly, even for difficult problems, checking behavior is associated with an increased probability of making a correct choice (Johnson et al., 2017). Thus, the ratio of trials with checking to total trials was compared across the different target-lure overlap conditions, sexes and experimental groups with a repeated measures ANOVA. Figure 5D shows the mean ratio of checking behavior as a function of lure similarity for the different groups. Checking behavior significantly decreased as a function of target-lure overlap (*F*_[3,78]_ = 33.79, *p* = 0.0001). Planned orthogonal difference contrasts indicated that while the 0 and 50% overlap conditions were not significantly different (*p* = 0.85), there was significantly less checking behavior during the 70% overlap condition compared to 50% (*p* = 0.0001). Moreover, animals also had significantly less checking for the 90% overlap condition relative to 70% (*p* = 0.0001). The amount of checking behavior as a function of target-lure overlap did not significantly interact with sex (*F*_[3,78]_ = 0.18, *p* = 0.91), or group (*F*_[3,78]_ = 1.78, *p* = 0.16), nor was the three-way interaction significant (*F*_[3,78]_ = 0.74, *p* = 0.53). The main effect of sex on checking behavior did not reach statistical significance (*F*_[1,6]_ = 1.53, *p* = 0.23). However, there was a trend for the PPT rats to display significantly less checking behavior compared to sham animals (*F*_[1,26]_ = 3.96, *p* = 0.057). Finally, the interaction between sex and group on total checking behavior did not reach statistical significance (*F*_[1,26]_ = 1.46, *p* = 0.24). Together these data suggest that unilateral PPT leads to an increased response bias as rats cannot disambiguate two similar stimuli and an associated reduction in checking behavior.

## Discussion

The present study tested the hypothesis that there is a causal relationship between perforant path fiber loss and a decreased ability to discriminate between stimuli that share features by unilaterally transecting the perforant path fibers of the right hemisphere in young male and female rats. We observed that unilaterally damaging the perforant path fibers, leads to a decreased ability of rats to choose a familiar target object over a similar lure containing overlapping features. Interestingly, the group differences between Sham and PPT animals was most evident for the testing problem in which target-lure similarity was greatest (90%, Figure 3B). While the PPT group was impaired at discriminating between similar objects, the fiber damage did not appear to produce a deficit in the rats’ abilities to remember the rewarded target. In fact, when the rats were trained to select a target object both prior to and following surgery, the PPT animals showed the same savings as the Sham group, retraining to criterion performance at the same rate (Figure 3A). Moreover, the PPT rats were able to benefit from experience with target-lure problems to a similar degree as the Sham animals by showing improved performance on all target-lure discrimination problems as a function of test day (Figure 4). Thus, the observed deficits in discrimination do not appear to be due to an inability to associate the target with the reward, but rather from a reduced ability to disambiguate between stimuli in which perceptual interference is high.

The finding that unilateral perforant fiber damage was sufficient to produce a behavioral deficit is consistent with previous studies showing that unilateral lesions to regions within the medial temporal lobe can produce impairments. Unilateral inactivation of the hippocampus in young rats impairs performance on the Morris watermaze test of spatial memory (Fenton and Bures, 1993; Cimadevilla et al., 2005), and leads to deficits on a spatial passive avoidance task (Lorenzini et al., 1996). Furthermore, older rats with damage to the left hippocampus make more spatial working memory errors on the 8-arm radial maze compared to animals with both hippocampi intact (Poe et al., 2000). In cortical structures, young rats with unilateral inactivation of the lateral entorhinal cortex are impaired at associating objects with contexts (Wilson et al., 2013a,b), and have deficits in the expression of eyeblink conditioning (Tanninen et al., 2013). These approaches that lesion gray matter not only affect the target region, however. When one hemisphere of the hippocampus or the entorhinal cortex is inactivated, back projections to sensory association cortices, the prefrontal cortex, as well as other regions of the limbic network are impacted. The novel aspect of the current study is that selectively disconnecting the parahippocampal region from the hippocampus in the right hemisphere, which leaves the back projections and other limbic white matter structures intact, induced a behavioral deficit. In should be noted, that while the fiber transection did not lead to a complete ablation of entorhinal cortical neurons (as evident in Figure 2C), it is conceivable that the damage to the axons produced a modest loss of neurons that was not qualitatively observable but still contributed to the behavioral deficits observed here.

**FIGURE 6:**
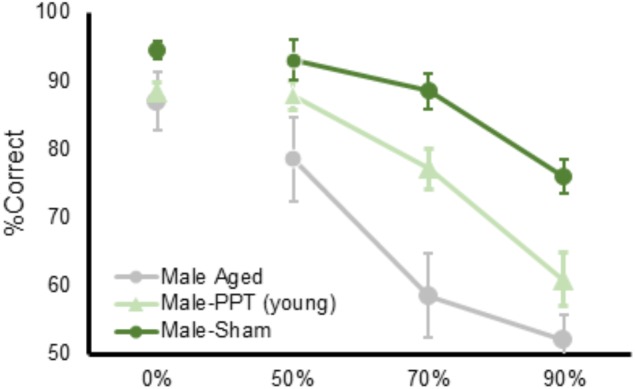
A comparison of the PPT rat mnemonic discrimination task performance compared to aged rats. Percent correct (*Y*-axis) as a function of target-lure similarity (*X*-axis) for rats from the current study plotted against young and aged rats tested in Johnson et al. (2017). The impairment following perforant path transection does not match the magnitude of the deficit previously observed in aged rats.

Aged rats, tested on the same apparatus used here by the same experimenters, are impaired at discriminating between objects that share features (Johnson et al., 2017). Remarkably, when the performances of aged rats from a previous study conducted in the same lab were compared to the current data, the deficit of the PPT rats was not of the same magnitude as what occurs in normal aging. Figure 6 shows the data from male rats in the current experiment plotted with data collected in aged male rats (24 months old) from Johnson et al. (2017); data from aged females are not available). From these data, it is qualitatively evident that the right side PPT does not impair performance to the same degree as observed in aged animals. This observation supports an emerging idea that although age-related perforant path fiber loss contributes to a diminished ability to discriminate between stimuli that have perceptual overlap, this is not likely the only locus of neurobiological dysfunction in older animals that contributes to behavioral deficits. In fact, high resolution diffusion tensor imaging data have shown that reduced white matter integrity of the hippocampal cingulum and the fornix of the hippocampus in older adults correlates with impaired discrimination of similar objects (Bennett et al., 2015; Bennett and Stark, 2016). Together these data suggest that the ability to disambiguate perceptually similar stimuli relies on the larger limbic network, which may become vulnerable in advanced age. In line with this idea, functional imaging studies have shown that BOLD levels in the thalamus, occipital areas, and prefrontal cortex are related to the ability to discriminate between perceptually similar stimuli (Pidgeon and Morcom, 2016; Reagh et al., 2017). The prefrontal cortex, which projects to the parahippocampal region through the cingulum, is vulnerable in advanced age (e.g., Barense et al., 2002; Nicolle and Baxter, 2003; Morrison and Baxter, 2012; Samson and Barnes, 2013; Banuelos et al., 2014; Chadick et al., 2014; Hernandez et al., 2017, 2018). Moreover, recent behavioral data suggest that the prefrontal cortex may be necessary for discriminating between similar stimuli. Specifically, when tested on a touch screen version of a trial-unique non-matching to location task, which simultaneously tests working memory and spatial discrimination (Talpos et al., 2010), NMDA receptor blockade in the prefrontal cortex impaired the discrimination of two similar locations (Davies et al., 2017). It is therefore conceivable that prefrontal-parahippocampal interactions through the cingulum are critical for disambiguating perceptually similar stimuli, although this idea needs to be empirically tested.

As noted in the *Introduction* section, we have elected to not frame the current data in terms of pattern separation deficits that are induced by perforant path fiber damage. It has been argued that the terminology used to describe behavioral measures of stimulus discrimination should be parsed from the computational processes that may orthogonalize similar input (Santoro, 2013). Rather than trying to infer computational processes from behavioral data, we contend that the parahippocampal region-hippocampal interactions that are involved in processing fine-grained “detailed” information necessary for disambiguating perceptually similar stimuli (Burke et al., 2018), are compromised by the unilateral PPT. Interestingly, the deficits observed in the current experiments are quite distinct from those seen when dentate gyrus activity is disrupted. In the latter case, animals show robust impairments in discriminating between the familiar target and a novel lure across all levels of similarity, but only when the lure is novel. After experience with the lure objects, rats with a disrupted dentate gyrus were able to perform the task comparable to control animals (Johnson et al., 2018). The deficit induced by the perforant path fiber transection may therefore be a result of reduced connectivity between the parahippocampal region and CA3 and/or CA1. Future experiments are needed to empirically test this idea.

## Author Contributions

SB designed the experiments, analyzed the data, and wrote the paper. ST collected and helped to analyze the data as well as helped to write the paper. CD collected and helped analyze the data. SJ helped design the experiments as well as analyze and interpret the data. AM designed the experiments, obtained funding, and wrote the manuscript.

## Conflict of Interest Statement

The authors declare that the research was conducted in the absence of any commercial or financial relationships that could be construed as a potential conflict of interest.
